# Genetic studies of complex human diseases: Characterizing SNP-disease associations using Bayesian networks

**DOI:** 10.1186/1752-0509-6-S3-S14

**Published:** 2012-12-17

**Authors:** Bing Han, Xue-wen Chen, Zohreh Talebizadeh, Hua Xu

**Affiliations:** 1 Bioinformatics and Computational Life-Sciences Laboratory, ITTC, Department of Electrical Engineering and Computer Science, University of Kansas, 1520 West 15th Street, Lawrence, KS 66045, USA; 2 Department of Computer Science Wayne State University Detroit, MI 48202; 3 Children's Mercy Hospital and University of Missouri-Kansas City School of Medicine, 2401 Gillham Road, Kansas City, MO 64108, USA; 4 School of Biomedical Informatics The University of Texas Health Science Center at Houston Houston, TX 77030

## Abstract

**Background:**

Detecting epistatic interactions plays a significant role in improving pathogenesis, prevention, diagnosis, and treatment of complex human diseases. Applying machine learning or statistical methods to epistatic interaction detection will encounter some common problems, e.g., very limited number of samples, an extremely high search space, a large number of false positives, and ways to measure the association between disease markers and the phenotype.

**Results:**

To address the problems of computational methods in epistatic interaction detection, we propose a score-based Bayesian network structure learning method, EpiBN, to detect epistatic interactions. We apply the proposed method to both simulated datasets and three real disease datasets. Experimental results on simulation data show that our method outperforms some other commonly-used methods in terms of power and sample-efficiency, and is especially suitable for detecting epistatic interactions with weak or no marginal effects. Furthermore, our method is scalable to real disease data.

**Conclusions:**

We propose a Bayesian network-based method, EpiBN, to detect epistatic interactions. In EpiBN, we develop a new scoring function, which can reflect higher-order epistatic interactions by estimating the model complexity from data, and apply a fast Branch-and-Bound algorithm to learn the structure of a two-layer Bayesian network containing only one target node. To make our method scalable to real data, we propose the use of a Markov chain Monte Carlo (MCMC) method to perform the screening process. Applications of the proposed method to some real GWAS (genome-wide association studies) datasets may provide helpful insights into understanding the genetic basis of Age-related Macular Degeneration, late-onset Alzheimer's disease, and autism.

## Background

To identify genetic variants that affect susceptibility of a variety of diseases, genome-wide association studies (GWAS) genotype a dense set of common SNPs (Single Nucleotide Polymorphism) and test allelic frequencies among a cohort of affected people and non-affected people [1]. Traditional analysis methods for GWAS data only consider one SNP at a time and test its association with disease. This type of analysis strategy is only suitable for simple Mendelian disorders. Some common complex diseases such as various types of cancers, cardiovascular disease, and diabetes are influenced by multiple genetic variants. Therefore, detecting high-order epistasis, which refers to the interactive effect of two or more genetic variants on complex human diseases, can help to unravel how genetic risk factors confer susceptibility to complex diseases [2]. However, the very large number of SNPs checked in a typical GWAS and the enormous number of possible SNP combinations make detecting high-order epistatic interactions from GWAS data computationally challenging [3]. Moreover, how to measure the association between a set of SNPs and the phenotype presents another grand statistical challenge.

During the past decade, two types of heuristic computational methods have been proposed to detect epistatic interactions: prediction/classification-based methods and association-based methods. Prediction/classification-based methods try to find the best set of SNPs, which can generate the highest prediction/classification accuracy including, for example, multifactor dimensionality reduction (MDR) [4], penalized logistic regression (e.g., stepPLR [5], and lassoPLR [6]), support vector machine (SVM) [7], and random forest [8]. MDR is a non-parametric and model-free method based on constructing a risk table for every SNP combination [4]. If the case and control ratio in a cell of this risk table is larger than 1, MDR will label it as "high risk", otherwise, "low risk". By the risk table, MDR can predict disease risk and will select the SNP combination with the highest prediction accuracy. StepPLR and lassoPLR make some modifications to avoid the overfitting problems that standard logistic regression methods suffer from [9] when detecting epistatic interactions. For example, stepPLR combines the logistic regression criterion with a penalization of the L2-norm of the coefficients. This modification makes stepPLR more robust to high-order epistatic interactions [5]. Two machine learning methods: SVM [7] and random forest [8] have also been applied to detecting epistatic interactions. Machine learning methods are based on binary classification (prediction) and treat cases as positives and controls as negatives in SNP data. They use SVM or random forest as a predictor and select a set of SNPs with the highest prediction/classification accuracy by feature selection. Some prediction/classification-based methods can only be applied to small-scale analysis (i.e., a small set of SNPs) due to their computational complexity. Moreover, almost all prediction/classification-based methods tend to introduce a large number of false positives, which may result in a huge cost for further biological validation experiments [10].

Bayesian epistasis association mapping (BEAM) is a scalable and association-based method [11]. It partitions SNPs into three groups: group 0 is for normal SNPs, group 1 contains disease SNPs affecting disease risk independently, and group 2 contains disease SNPs that jointly contribute to the disease risk (interactions). Given a fixed partition, BEAM can get the posterior probability of this partition from SNP data based on Bayesian theory. A Markov Chain Monte Carlo method is used to reach the optimal SNP partition with maximum posterior probability in BEAM. One drawback of BEAM is that identifying both single disease SNP and SNP combinations simultaneously makes BEAM over-complex and weakens its power.

Recently, we propose a new Markov blanket-based method, DASSO-MB, to detect epistatic interactions in case-control studies [10]. The Markov Blanket is a minimal set of variables, which can completely shield the target variable from all other variables based on Markov condition property [12]. Thus, Markov blanket methods can detect the causal disease SNPs with the fewest false positives. Furthermore, the heuristic search strategy in Markov blanket methods can avoid the time-consuming training process as in SVM and random forests. However, the faithfulness assumption in Markov blanket methods, which can hardly always be ensured, may hinder their applications in detecting epistatic interactions [13].

In this paper, we address the two critical challenges (small sample sizes and high dimensionality) in epistatic interaction detection by introducing a score-based Bayesian network structure learning method, EpiBN (Epistatic interaction detection using Bayesian Network model), which employs a Branch-and-Bound technique and a new scoring function. Bayesian networks provide a succinct representation of the joint probability distribution and conditional independence among a set of variables. In general, a score-based structure learning method for Bayesian networks first defines a scoring function reflecting the fitness between each possible structure and the observed data, and then searches for a structure with the maximum score. Comparing to Markov blanket methods, the merits of applying score-based Bayesian network structure learning method to epistatic interaction detection include: (1) the faithfulness assumption can be relaxed and (2) heuristic search method can solve the classical XOR (Exclusive or) problem [14]. We apply the EpiBN method to simulated datasets based on four disease models and three real datasets: Age-related Macular Degeneration (AMD) dataset, late-onset Alzheimer's disease (LOAD) dataset, and autism dataset. We demonstrate that the proposed method outperforms some commonly-used methods such as SVM, MDR, and BEAM, especially when the number of samples is small.

## Methods

### Bayesian networks: a brief introduction

A Bayesian network is a directed acyclic graph (DAG) *G *consisting of nodes corresponding to a random variable set {*X*_1_, *X*_2_, ..., *X_n_*} and edges between nodes, which determine the structure of *G *and therefore the joint probability distribution of the whole network [15]. For three random variables (nodes) *X, Y *and *Z*, if the probability distribution of *X *conditioned on both *Y *and *Z *is equal to the probability distribution of *X *conditioned only on *Y*, i.e., *P*(*X*|*Y, Z*) = *P*(*X*|*Y*), *X *is conditionally independent of *Z *given *Y*. This conditional independence is represented as(X ⊥ *Z *| *Y*) [16]. The DAG *G *encodes local Markov assumption: each variable is conditionally independent of its nondescendants, given its parents in *G*. By applying the local Markov assumption, the joint probability distribution *J *can be represented as

(1) $$P\left(X_{1},...,X_{n}\right)=\overset{n}{\underset{i=1}{\prod }}P\left(X_{i}|Pa\left(X_{i}\right)\right)$$

where *Pa*(*X_i_*) denotes the set of parents of *X_i _*in *G*. Therefore, there are two components in a Bayesian network. The first component is the DAG *G *reflecting the structure of the network. The second component, *θ*, describes the conditional probability distribution *P*(*X_i_*|*Pa*(*X_i_*)) to specify the unique distribution *J *on *G*.

Bayesian networks provide models of causal influence and allow us to explore causal relationships, perform explanatory analysis, and make predictions. Genome-wide association studies attempt to identify the epistatic interaction among a set of SNPs, which are associated with one certain type of disease. Therefore, we can use Bayesian networks to represent the relationship between genetic variants and a phenotype (disease status). The *n *SNP nodes and the disease status/label node form a two-layer Bayesian network and we want to determine which SNP nodes are the parent nodes of the disease status node. In this type of Bayesian network, we only allow edges from SNP nodes to the disease status node. Edges from the disease status node to SNP nodes and edges among SNP nodes are prohibited.

By modelling the association between SNPs and the disease status based on Bayesian networks, we transform detecting epistatic interactions into structure learning of Bayesian networks from GWAS data. There are two types of structure learning methods for Bayesian networks: constraint-based methods and score-and-search methods. The constraint-based methods first build a skeleton of the network (undirected graph) by a set of dependence and independence relationships. Next they direct links in the undirected graph to construct a directed graph with *d*-separation properties corresponding to the dependence and independence determined [17,18]. Although constraint-based methods are developed with a rigorous theoretical foundation, errors in conditional dependence and independence will affect the stability of constraint-based methods, especially for small sample problems, which is also a problem of Markov Blanket methods in detecting epistatic interactions. Therefore, in this paper, we focus on score-and-search methods. The score-and-search methods view a Bayesian network as a statistical model and transform the structure learning of Bayesian networks into a model selection problem [19]. To select the best model, a scoring function is needed to indicate the fitness between a network and the data. Then the learning task is to find the network with the highest score. Thus, score-and-search methods typically consist of two components, (1) a scoring function, and (2) a search procedure. Next, we discuss in detail the proposed EpiBN algorithm, which consists of three components: scoring, searching, and screening.

### EpiBN scoring: A new BN scoring function

One of the most important issues in score-and-search methods is the selection of scoring function. A natural choice of scoring function is the likelihood function. However, the maximum likelihood score often overfits the data because it does not reflect the model complexity. Therefore, a good scoring function for Bayesian networks' structure learning must have the capability of balancing between the fitness and the complexity of a selected structure. There are several existing scoring functions based on a variety of principles, such as the information theory and minimum description length (e.g. BIC score, AIC score, and MDL score) [20-22] and Bayesian approach (BDe score) [23].

Suppose that a dataset D includes *n *variables {*X*_1_, *X*_2_, ..., *X_n_*} and *N *samples, we can write a general information-based scoring function as:

(2) $$\text{log}P\left(D|S\right)=\text{log}P\left(D|{\hat{\theta }}_{S},S\right)-C\left(S\right)f\left(N\right)$$

(3) $$C\left(S\right)=\overset{n}{\underset{i=1}{\sum }}q_{i}\left(r_{i}-1\right)$$

where ${\hat{\theta }}_{S}$is an estimate of parameters from the maximum likelihood method for the structure S, *q_i _*is the number of configurations of the parent set *Pa*(*X_i_*) of *X_i_, r_i _*is the number of states of *X_i_, C*(*S*) represents the structure complexity, and *f*(*N*) is a penalization function [24]. The first term of this score scheme measures the fitness between the structure and data, and the second term reflects structure complexity. With the maximum likelihood method [19], we can get

(4) $$\text{log}\left(P\left(D|{\hat{\theta }}_{S},S\right)\right)=\overset{n}{\underset{i=1}{\sum }}\overset{q_{i}}{\underset{j=1}{\sum }}\overset{r_{i}}{\underset{k=1}{\sum }}N_{ijk}\text{log}\left(N_{ijk}/N_{ij}\right)$$

where *N_ijk _*is the number of instances where *X_i _*takes its *k*-th value and the set of variables *Pa*(*X_i_*) takes its *j*-th configuration; *N_ij _*is the number of instances where the set of variables *Pa*(*X_i_*) takes its *j*-th configuration. Obviously, $N_{ij}=\sum_{k=1}^{r_{i}}N_{ijk}$. Note that if we set *f*(*N*) = 1, we get the AIC score as

(5) $$\text{log}P\left(D|S\right)=\text{log}P\left(D|{\hat{\theta }}_{S},S\right)-C\left(S\right)$$

Alternatively, if we set *f*(*N*) = 1/2 log(*N*), then we obtain the BIC score as

(6) $$\text{log}P\left(D|S\right)=\text{log}P\left(D|{\hat{\theta }}_{S},S\right)-1/2C\left(S\right)\text{log}\left(N\right)$$

The BIC score and AIC score are derived from some approximations when the number of samples *N *approaches infinity [25]. If the number of samples is small, the approximation in the inference of both AIC and BIC scores can not hold any more and the structure penalty term in Eq. (5) and Eq. (6) are not suitable [26]. When using information-based scores in the Bayesian network model to detect epistatic interactions by GWAS data, which show a non-skewed distribution, the BIC score is too strict and prefers to select simple structures, while the AIC score prefers to select complex structures [27].

We herein describe a new information-based scoring function to detect epistatic interactions by Bayesian network model. In the Bayesian network for epistatic interaction detection, we are only concerned with one target node, the disease status node, and we want to detect its parent SNP nodes. We represent the local structure around the disease status node as *LDS *(Local Disease Structure), which consists of the disease status node and edges from candidate disease SNP nodes to the disease status node. Because of the decomposability property of information-based scoring function, the AIC score for *LDS *is:

(7) $$\begin{array}{c} \text{log}P\left(D|LDS\right)=\text{log}P\left(D|{\hat{\theta }}_{LDS},LDS\right)-C\left(LDS\right)\\ =\overset{q}{\underset{j=1}{\sum }}\overset{r}{\underset{k=1}{\sum }}N_{jk}\text{log}\left(N_{jk}/N_{j}\right)-q\left(r-1\right) \end{array}$$

where *C*(*LDS*) is the complexity of the local disease structure, *q *is the number of configurations of parent SNP nodes, *r *is the number of states of the disease status node, *N_jk _*is the number of instances where the disease status node takes its *k*-th value and the parent SNP nodes take their *j*-th configuration, *N_j _*is the number of instances where the parent SNP nodes take their *j*-th configuration, and $N_{j}=\sum_{k=1}^{r}N_{jk}$.

We start our search from an empty local disease structure *LDS*_0_, and we can obtain the AIC score for *LDS*_0_:

(8) $$\begin{array}{c} \text{log}P\left(D|LDS_{0}\right)=\text{log}P\left(D|{\hat{\theta }}_{LDS_{0}},LDS_{0}\right)-C\left(LDS_{0}\right)\\ =\overset{r}{\underset{k=1}{\sum }}N_{k}\text{log}\left(N_{k}/N\right)-\left(r-1\right) \end{array}$$

where *N_k _*is the number of instances in which the disease status node takes its *k*-th value, and $N=\sum_{k=1}^{r}N_{k}$.

For further inference, we use *X *for the target disease status node and use *Pa*(*X*) for its parent SNP nodes. The log-likelihood of *LDS *and *LDS*_0 _can also be expressed as follows:

(9) $$\text{log}P\left(D|{\hat{\theta }}_{LDS},LDS\right)=-N*H\left(X|Pa\left(X\right)\right)$$

(10) $$\text{log}P\left(D|{\hat{\theta }}_{LDS_{0}},LDS_{0}\right)=-N*H\left(X\right))$$

where *H*(*X*) is the entropy of *X *and *H*(*X*|*Pa*(*X*)) is the conditional entropy of *X *given its parent set *Pa*(*X*) [28]. Based on the concept of mutual information and Eq. (7)-(10), the mutual information between *X *and *Pa*(*X*) is:

(11) $$\begin{array}{c} MI\left(X,Pa\left(X\right)\right)=H\left(X\right)-H\left(X|Pa\left(X\right)\right)\\ =\frac{\text{log}P\left(D|{\hat{\theta }}_{LDS},LDS\right)-\text{log}P\left(D|{\hat{\theta }}_{LDS_{0}},LDS_{0}\right)}{N} \end{array}$$

i.e. the mutual information between *X *and *Pa*(*X*) coincides with the difference between the log-likelihood of *LDS *and *LDS*_0 _[24].

The *G*^2 ^test is commonly used to test independence and conditional independence between two variables for discrete data. From the general formula for *G*^2^, we know that the value of *G*^2 ^can also be calculated from mutual information [29]. Thus, we can write the *G*^2 ^test value between *X *and *Pa*(*X*) as:

(12) $$G^{2}\left(X,Pa\left(X\right)\right)=2N\left(MI\left(X,Pa\left(X\right)\right)\right)=2N\left(H\left(X\right)-H\left(X|Pa\left(X\right)\right)\right)$$

The number of degrees of freedom for *G*^2 ^test between *X *and *Pa*(*X*) is:

(13) $$\begin{array}{c} DF\left(G^{2}\left(X,Pa\left(X\right)\right)\right)=\left(Cat\left(X\right)-1\right)\left(Cat\left(Pa\left(X\right)\right)-1\right)\\ =\left(r-1\right)\left(q-1\right) \end{array}$$

where *Cat*(*V*) is the number of categories of the variable *V*, and thus *Cat*(*X*) = *r *and *Cat*(*Pa*(*X*)) = *q *[18].

It is interesting to note that the difference between the complexity of *LDS *and *LDS*_0 _is equal to the degree of freedom of *G*^2^(*X, Pa*(*X*)) by

(14) $$\begin{array}{c} C\left(LDS\right)-C\left(LDS_{0}\right)=\left(r-1\right)q-\left(r-1\right)\\ =\left(r-1\right)\left(q-1\right)=DF\left(G^{2}\left(X,Pa\left(X\right)\right)\right) \end{array}$$

By applying Eq. (7)-(14), the difference of AIC scores between *LDS *and *LDS*_0 _is:

(15) $$\begin{array}{c} \text{log}P\left(D|LDS\right)-\text{log}P\left(D|LDS_{0}\right)\\ =N(MI\left(X,Pa\left(X\right)\right)-\left(r-1\right)\left(q-1\right)\\ =1/2\left(G^{2}\left(X,Pa\left(X\right)\right)-2DF\left(G^{2}\left(X,Pa\left(X\right)\right)\right)\right) \end{array}$$

Thus, the AIC score becomes:

(16) $$\begin{array}{c} \text{log}P\left(D|LDS\right)\\ =1/2\left(G^{2}\left(X,Pa\left(X\right)\right)-2DF\left(G^{2}\left(X,Pa\left(X\right)\right)\right)\right)+\text{log}P\left(D|LDS_{0}\right) \end{array}$$

where log *P*(*D*|*LDS*_0_) is a constant.

The distribution of *G*^2 ^asymptotically approximates to that of *χ*^2 ^with the same number of degrees of freedom [18]. The *χ*^2 ^distribution with *k *degrees of freedom has a variance of *2k*, and therefore 2*DF*(*G*^2^(*X, Pa*(*X*))) is the variance of the corresponding *G*^2 ^distribution. Since *G*^2^(*X, Pa*(*X*)) reflects the bias, the AIC score in Eq. (16) indicates a trade-off between bias and variance in terms of the *G*^2 ^statistic *G*^2^(*X, Pa*(*X*)) and its variance.

One problem for the AIC score in Eq. (5), Eq. (7), and Eq. (16) is that the penalty term (the effective number of parameters) in AIC score probably can not reflect the model complexity (or variance) especially when applied to SNP data with a non-skewed distribution. We can confirm this by comparing 2*DF*(*G*^2^(*X, Pa*(*X*))) with the true variance of *G*^2^(*X, Pa*(*X*)) from SNP data. There is a large deviation between them when *Pa*(*X*) contains more than two parent nodes. The more parent nodes *Pa*(*X*) contains, the larger the deviation is because of the increasing model complexity and hence the increasing 'difficulty in estimation' [30]. One simple but practical way to consider and estimate the model complexity in AIC score is replacing 2*DF*(*G*^2^(*X, Pa*(*X*))) in Eq. (16) with the true variance of *G*^2^(*X, Pa*(*X*)) from data and our new epistatic scoring function (EpiScore) becomes:

(17) $$\begin{array}{c} EpiScore\left(LDS:D\right)=\text{log}P\left(D|LDS\right)\\ =1/2\left(G^{2}\left(X,Pa\left(X\right)\right)-Var_{D}\left(G^{2}\left(X,Pa\left(X\right)\right)\right)\right)+\text{log}P\left(D|LDS_{0}\right) \end{array}$$

where *Var_D_*(*G*^2^(*X, Pa*(*X*))) comes from the estimation of the variance of the corresponding *G*^2 ^distribution from data. Our new scoring function estimates the penalty term from the data to make it consistent with the data, which is similar to the DIC (Deviance Information Criterion) score trying to identify models that best explain the observed data [30].

Due to the estimation of the variance of *G*^2^(*X, Pa*(*X*)) from data in Eq. (17), EpiScore is not score-equivalent. However, we are not very concerned about this: there are no equivalent structures in the two-layer Bayesian network for the restriction on the direction of edges we describe in the previous section.

### EpiBN searching: A Branch-and-Bound algorithm for local structure learning

The computational task in score-and-search methods is to find a network structure with the highest score. The searching space consists of a super-exponential number of structures and thus exhaustively searching optimal structure from data for Bayesian networks is NP-hard [31]. One simple heuristic search algorithm is greedy hill-climbing algorithm, where three types of operators are defined to change one edge at each step: adding an edge, removing an edge, and reversing an edge. By these three operators, we can construct the local neighbourhood of the current network. Then we select the network with the highest score in the local neighbourhood to get the maximal gain. This process can be repeated until it reaches a local maximum. However, greedy hill-climbing algorithm cannot guarantee a global maximum [19]. Other structure learning methods for Bayesian networks include Branch-and-Bound (B&B) [28,32] and Markov chain Monte Carlo [33].

**Figure 1:**
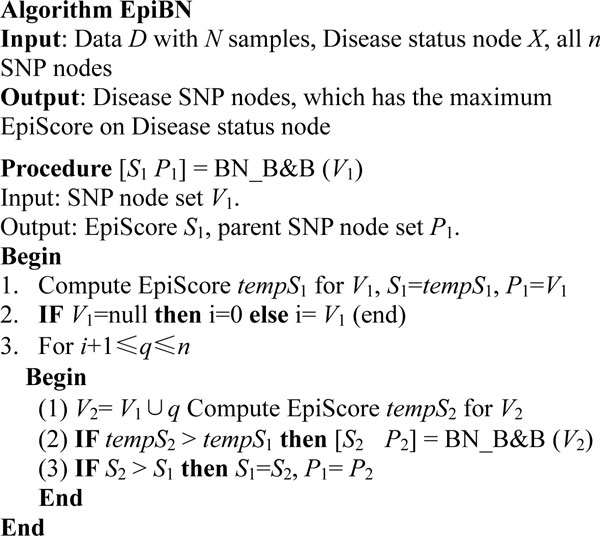
**EpiBN Algorithm**.

We employ B&B algorithm in our study because the B&B algorithm can guarantee the optimal results in a significantly reduced search time compared to exhaustive search. Our EpiBN method uses B&B algorithm to search a local disease structure that maximizes the EpiScore in Eq. (17). The pseudo code of EpiBN is shown in Figure 1. In EpiBN, the procedure BN_B&B starts from an empty parent node set and constructs a depth-first search tree to find the optimal parent (disease SNPs) set for the disease status node. In our B&B search, instead of using the pruning strategy as in [28], which sets a lower bound for the MDL score to prune the search tree, we stop the recursive calls when we observe that the score will decrease on the children state of the current state. This strategy cannot guarantee global optima theoretically. However, it will significantly speed up the search process and perform well practically.

To guarantee to find the parent set with the highest EpiScore, we can use the upper bound of the EpiScore to prune the search tree. We notice the *G*^2 ^function in Eq. (12) has the property:

(18) $$0\leq G^{2}\left(X,Pa\left(X\right)\right)=2N\left(H\left(X\right)-H\left(X|Pa\left(X\right)\right)\right)\leq 2N*H\left(X\right)$$

When adding a SNP node *q *into the parent set *V*_1_, the variance of the corresponding *G*^2 ^distribution, the penalty term in Eq. (17), will increase by *Var_D_*(*G*^2^(*X, V*_2_)) - *Var_D_*(*G*^2^(*X, V*_1_)). On the other hand, the *G*^2^(*X, V*_1_) will increase at most by 2N*H(*X*) because the value of entropy *H*(*X*|*Pa*(*X*)) will decrease and is nonnegative. Hence, if we find

(19) $$Var_{D}\left(G^{2}\left(X,V_{2}\right)\right)-Var_{D}\left(G^{2}\left(X,V_{1}\right)\right)>2N*H\left(X\right)$$

adding a SNP node *q *into the current parent set *V*_1 _will not increase the EpiScore and thus any further search along the branch is useless. Essentially, the upper bound of the EpiScore is

(20) $$EpiScore\left(LDS:D\right)=\text{log}P\left(D|LDS\right)\leq N*H\left(X\right)+\text{log}P\left(D|LDS_{0}\right)$$

### EpiBN screening: MCMC screening method for real datasets

Even though the B&B algorithm uses an upper score bound to reduce the searching space, it still has an exponential time complexity in the worst case and is not feasible to be directly applied to real GWAS data. Therefore, an efficient screening method is necessary. Traditional screening methods assign a score to every single SNP and select a subset of SNPs with high scores. However, these methods ignore the joint effect of SNPs on disease and are not suitable for detecting epistatic interactions from real GWAS data.

In this paper, we use the Markov chain Monte Carlo (MCMC) method [33] to perform the screening process. In the Bayesian network for epistatic interaction detection, we use a Metropolis-Hastings method to build a Markov chain to get the posterior probability for each edge from the SNP nodes to the disease status node. At each step of the Markov chain, we use two types of moves: add an edge and remove an edge. The proposed move is accepted with probability

(21) $$\alpha =\text{min}\left\{1,R_{\alpha }\right\}$$

where

(22) $$R_{\alpha }=\frac{\#\left(nbd\left(LDS\right)\right)P\left(LDS^{\prime }|D\right)}{\#\left(nbd\left(LDS^{\prime }\right)\right)P\left(LDS|D\right)}$$

where #(*nbd*(*LDS*)) is the cardinality of the neighbourhood of the current local disease structure and *LDS' *is the candidate local disease structure in each step of the Markov chain. Since *LDS *and *LDS' *differ in one move, the ratio #(*nbd*(*LDS*))/#(*nbd*(*LDS'*)) is one. In addition, the posterior probability of the local disease structure, *P*(*LDS*|*D*), is that *P*(*LDS*|*D*) ∝ *P*(*D*|*LDS*) *P*(*LDS*) and we take a uniform distribution over the considered local disease structures. Therefore, the acceptance ratio in Eq. (22) becomes:

(23) $$R_{\alpha }=P\left(D|LDS^{\prime }\right)/P\left(D|LDS\right)$$

The likelihood of local disease structure, *P*(*D*|*LDS*), can be calculated by Eq. (17). Based on the result from MCMC method, we select SNP nodes associated with edges whose posterior probabilities larger than 0. Since we consider the association of multiple SNPs with disease status at each step of the Markov chain in our MCMC method, the potential disease SNPs related with epistatic interactions will be kept in the final subset of SNPs.

## Results

### Analysis of Simulated Data

*Simulated Data *We first evaluate the proposed EpiBN method on four simulated data sets, which are generated from three commonly used two-locus epistatic models in [9] and one three-locus epistatic model developed in [11]. Model-1 is a multiplicative model, model-2 demonstrates two-locus interaction multiplicative effects, and model-3 specifies two-locus interaction threshold effects. There are three disease loci in model-4 [11]. Some certain genotype combinations can increase disease risk in model-4 and there are almost no marginal effects for each disease locus.

We generate data based on the similar parameter settings as in [9-11] for three parameters associated with each model: the marginal effect of each disease locus (*λ*), the minor allele frequencies (MAF) of both disease loci, and the strength of linkage disequilibrium (LD, quantified by the squared correlation coefficient *r*^2 ^calculated from allele frequencies) between the unobserved disease locus and a genotyped locus [9]. For each parameter setting on each model, we generate 50 datasets and each dataset contains 100 markers genotyped for 1,000 cases and 1,000 controls. To measure the performance of each method, we use power as our evaluation criterion, which is defined as the proportion of simulated datasets in which only the true diseases associated markers are identified without any false positives.

**Figure 2:**
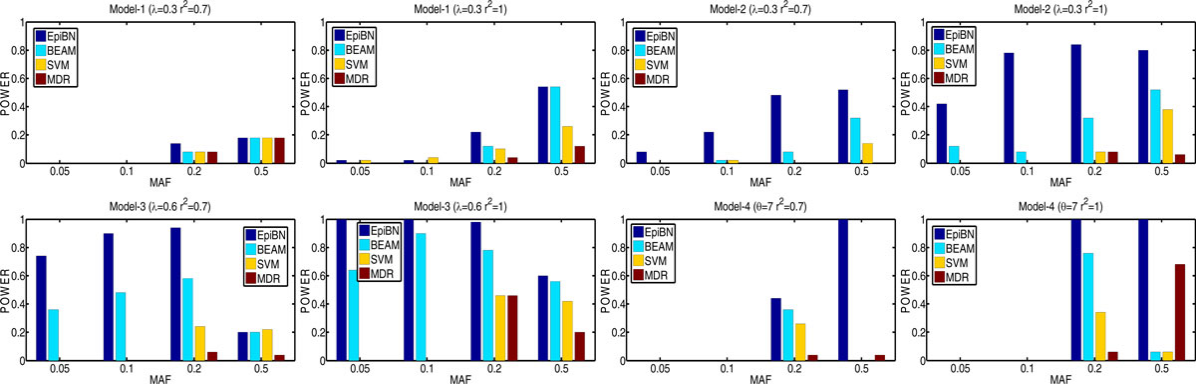
**Performance comparison of EpiBN, BEAM, SVM, and MDR**.

*EpiBN versus BEAM, SVM, and MDR *We first compare EpiBN with three methods: BEAM, SVM, and MDR on the four simulated disease models. The BEAM software is downloaded from http://www.fas.harvard.edu/~junliu/BEAM and we set the threshold of the B statistic as 0.1 [11]. For SVM, we use LIBSVM with a RBF kernel to detect gene-gene interactions. A grid search is used for selecting optimal parameters. Instead of using the exhaustive greedy search strategy for SNPs as in [7], which is very time-consuming and infeasible to large-scale datasets, we turn to a search strategy used in [8]. First we rank SNPs based on the mutual information between SNPs and disease status label that is 0 for the control and 1 for the case. Then, we use a sliding window sequential forward feature selection (SWSFS) algorithm in [8] based on SNPs rank. The window size in SWSFS algorithm determines how robust the algorithm could be and we set it to 20. Since MDR algorithm can not be applied to a large dataset directly, we first reduce the number of SNPs to 10 by ReliefF [34], a commonly-used feature selection algorithm, and then MDR performs an exhaustive search for a SNP set that can maximize cross-validation consistency and prediction accuracy. When one model has the maximal cross-validation consistency and another model has the maximal prediction accuracy, MDR follows statistical parsimony (selects the model with fewer SNPs). Our EpiBN is written in Matlab. The results on the simulated data are shown in Figure 2. As can be seen, among the four methods, the EpiBN method performs the best, and BEAM is the second best. One possible reason is that BEAM tries to detect single disease locus and epistatic interactions simultaneously. This strategy makes BEAM unnecessarily over-complex. In most cases, the powers of both MDR and SVM are much smaller than those of the EpiBN and BEAM algorithms.

Our definition of power prohibits any false positives and any false negatives and reflects the ability to precisely detect whole interactions [35]. Although we consider both type I error and type II error and the performance comparison is fair for each method in Figure 2, this type of definition of power seems stringent. To explore both false positive rates and false negative rates further, we measure the detection accuracy by precision and recall. Precision is the number of true disease SNPs in the output divided by the number of detected SNPs in the output, which reflects the false positive rate. On the other hand, recall is the number of true disease SNPs in the output divided by the number of true known disease SNPs, which reflects the false negative rate. The Euclidean distance from perfect precision and recall is defined as:

(24) $$\sqrt{{\left(1-precision\right)}^{2}+{\left(1-recall\right)}^{2}}$$

which combines precision and recall [36]. Table 1 presents the average precision, recall, and distance performance about EpiBN, BEAM, and SVM on 50 datasets for each model with MAF = 0.5 and *r*^2 ^= 1. EpiBN achieves a higher overall accuracy than both BEAM and SVM on model-2, model-3, and model-4. Moreover, the overall accuracy of EpiBN on model-4 is perfect. On model-1, EpiBN is still better than SVM while it is slightly worse than BEAM. BEAM shows the highest precision on the first three models, but it intends to miss more true positives. On the contrary, SVM demonstrates the highest recall, however, at the cost of introducing more false positives [10]. We do not evaluate the accuracy of MDR because the MDR software can only test at most 4-way epistatic interactions.

**Table 1:** Accuracy comparison of EpiBN, BEAM, and SVM.

Model	Method	Precision	Recall	Distance
1	EpiBN	0.76 ± 0.27	0.76 ± 0.27	0.34 ± 0.38
	
	BEAM	0.87 ± 0.32	0.75 ± 0.34	**0.32 ± 0.43**
	
	SVM	0.61 ± 0.29	0.91 ± 0.19	0.43 ± 0.31

2	EpiBN	0.90 ± 0.21	0.90 ± 0.20	**0.14 ± 0.29**
	
	BEAM	0.91 ± 0.26	0.75 ± 0.31	0.29 ± 0.38
	
	SVM	0.69 ± 0.29	0.95 ± 0.15	0.34 ± 0.31

3	EpiBN	0.78 ± 0.30	0.79 ± 0.30	**0.31 ± 0.43**
	
	BEAM	0.83 ± 0.35	0.74 ± 0.37	0.34 ± 0.49
	
	SVM	0.72 ± 0.28	0.88 ± 0.24	0.33 ± 0.35

4	EpiBN	1.00 ± 0.00	1.00 ± 0.00	**0.00 ± 0.00**
	
	BEAM	0.41 ± 0.49	0.20 ± 0.29	1.05 ± 0.47
	
	SVM	0.41 ± 0.32	0.61 ± 0.38	0.76 ± 0.40

*EpiScore versus BIC score and AIC score *We also compare our EpiScore with BIC score and AIC score. For BIC score and AIC score, we use the upper bound of score in [28] and the same B&B algorithm as in EpiBN. Table 2 presents the results on datasets with MAF = 0.5 and *r*^2 ^= 1. Column "o" shows the times of correct detection of all disease SNPs in 50 datasets. Column "+" presents the total number of extra detected SNPs and column "-" has the total number of missing disease SNPs. For model-1, mode-2, and model-3, EpiScore performs better than both BIC score and AIC score. BIC score can not detect true disease SNPs at all and introduce many false negatives due to its heavy penalty term. Comparing to EpiScore, AIC score tends to introduce more false positives and more false negatives. It is interesting to notice that every score function can achieve perfect power on model-4. The reason is that the relatively large genotypic effect, *θ*, in model-4 can generate data with skewed distribution, which can help all scoring function detect true disease SNPs.

**Table 2:** Comparison of EpiScore, BIC score, and AIC score.

Model	Score	o	+	-
1	EpiScore	27	24	24
	
	BIC score	0	0	57
	
	AIC score	12	55	31

2	EpiScore	40	11	10
	
	BIC score	40	11	10
	
	AIC score	22	36	14

3	EpiScore	30	23	21
	
	BIC score	0	0	57
	
	AIC score	10	53	20

4	EpiScore	50	0	0
	
	BIC score	50	0	0
	
	AIC score	50	0	0

"o": times of correct detection of all disease SNPs in 50 datasets. "+": total number of extra detected SNPs in 50 datasets. "-": total number of missing disease SNPs in 50 datasets.

**Figure 3:**
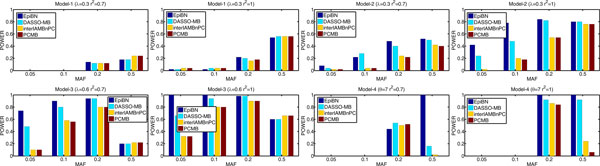
**Performance comparison of EpiBN with three Markov Blanket methods: interIAMBnPC, PCMB, and DASSO-MB**.

*EpiBN versus Markov Blanket methods *To demonstrate the advantages of EpiBN over Markov Blanket methods, we compare EpiBN with three Markov Blanket methods: interIAMBnPC [12], PCMB [36], and our DASSO-MB [10]. For interIAMBnPC, we use the Matlab toolbox Causal Explorer which contains the interIAMBnPC algorithm [37]. We implement both PCMB and DASSO-MB in Matlab. *G*^2 ^test is used to test dependence and independence in these three Markov Blanket methods and we set the p-value threshold for *G*^2 ^test as 0.01. Figure 3 shows the results. These four methods demonstrate the similar performance on the multiplicative model: model-1. On the other three interaction models: model-2, model-3, and model-4, EpiBN is better than these three Markov Blanket methods. Disease SNPs in model-1 affects the disease risk independently, which makes it easy for Markov Blanket methods to detect them. Additionally, DASSO-MB is better than the other two Markov Blanket methods: interIAMBnPC and PCMB. This is because the backward phase in DASSO-MB to remove false positives is not that strict as in interIAMBnPC and PCMB. Hence, DASSO-MB can keep SNP nodes having strong joint effects on disease status but not showing strong marginal effects in the Markov Blanket. This also confirms that the faithfulness assumption may hinder the application of Markov Blanket methods in detecting epistatic interactions.

*Sample efficiency *Typically, GWAS can not generate a large number of samples due to the high experiment cost. Thus, the performance of various computational methods for epistatic interaction detection in case of small samples is important. We explore the effect of the number of samples on the performance of EpiBN, MDR, BEAM and SVM. The parameters used are: *λ *= 1.1 for model-1, *λ *= 0.9 for model-2, *λ *= 1.8 for model-3, and *θ *= 7 for model-4. To test the scalability of EpiBN on large number of SNPs, we generate synthetic datasets containing different number of SNPs (40, 200, and 1000) genotyped for different number of samples (100, 200, 300, 400, 600, 1000, and 2000) with *r*^2 ^= 1 and MAF = 0.5.

**Figure 4:**
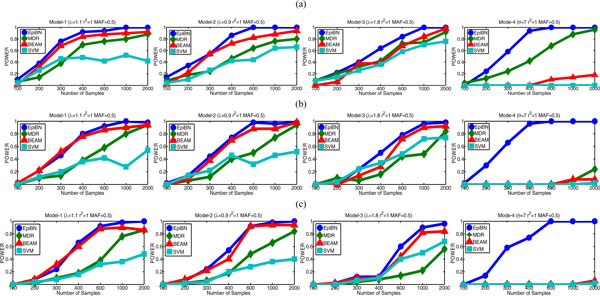
**Comparison of sample efficiency on datasets with different number of SNPs: (a) 40 SNPs, (b) 200 SNPs and (c) 1000 SNPs**.

The results are shown in Figure 4. We find that EpiBN is more sample-efficient than other methods in that it can achieve the highest power when the number of samples is the same. In addition, it needs fewer samples to reach the perfect power comparing to other methods. BEAM is still the second best. The powers of both MDR and SVM are still smaller than those of the EpiBN and BEAM algorithms. However, MDR and SVM demonstrate a better performance comparing to the results in Figure 2. This is perhaps due to the fact that increasing the marginal effect size *λ *for model-1, model-2, and model-3 makes the detecting task suitable for the pre-filtering based methods such as MDR and SVM. The result from model-4 is particularly interesting: EpiBN exhibits overwhelming superiority over other three methods, as EpiBN yields a perfect power even the number of samples is small (around 600), which indicates that EpiBN is especially suitable for detecting epistatic interactions with weak or no marginal effects. From Figure 4, we can also find that increasing the number of genotyping markers, like adding some noise to the data, will impair the power of all methods, especially in case of small samples.

### Analysis of AMD Data

In this section and the following two sections, we apply EpiBN to large-scale datasets in real genome-wide case-control studies, which often require genotyping of 30,000-1,000,000 common SNPs. We first make use of an Age-related Macular Degeneration (AMD) dataset containing 116,204 SNPs genotyped with 96 cases and 50 controls [38] (i.e., high dimensionality and small sample sizes). Multiple genetic factors cause AMD, which is a complex retinal degenerative disorder. To remove inconsistently genotyped SNPs, we perform the same filtering process as in [11,38]. After filtering, there are 97,327 autosomal SNPs remained.

We first perform the screening process and select 51 potential disease SNPs related with AMD by MCMC method. Among these 51 selected SNPs, EpiBN detects two associated SNPs showing the highest EpiScore: rs380390 and rs2402053. Klein *et al*. demonstrated that the first SNP, rs380390, is associated with AMD [38]. The second SNP, rs2402053, is intergenic between TFEC and TES in chromosome 7q31 [39]. Even though no evidences show that rs2402053 is related with AMD, it is worth noting that mutations in some genes on 7q31-q32 are revealed in patients with retinal disorders [40]. Therefore, rs2402053 may be a new genetic factor, on chromosome 7q, contributing to the underlying mechanism of AMD.

### Analysis of LOAD Data

Late-onset Alzheimer's disease (LOAD) is the most common form of Alzheimer's disease and usually occurs in persons over 65. It causes patients' degeneration of the ability of thinking, memory, and behaviour. The apolipoprotein E (APOE) gene is one genetic factor that accounts for affecting the risk of LOAD. There are three common variants of the APOE gene: *ε*2, *ε*3, and *ε*4. The appearance of the *ε*4 allele in a person's APOE genotype increases the LOAD risk [41]. Rieman *et al*. conducted genome-wide association studies to detect other generic risk factors related with LOAD [41]. They found 10 SNPs showing significant association with LOAD in the APOE *ε*4 carriers. All these 10 SNPs are in the GRB-associated binding protein 2 (GAB2) gene.

We download the LOAD GWAS data from http://www.tgen.org/neurogenomics/data. After pre-processing, we have 287,479 SNPs and 1408 samples (857 cases and 551 controls). EpiBN keeps APOE as one parent of the disease status node and identifies two other SNPs: rs1931565 and rs4505578, which may interact with APOE and affect the LOAD risk. The rs1931565 SNP is intergenic between ABCA4 and ARHGAP29 in chromosome 1p22. ABCA4 is related with some brain-related diseases including stargardt disease 1, early-onset severe retinal dystrophy and age-related macular degeneration [42]. On the other hand, some ABC transporter family genes such as ABCA1, ABCA2, ABCA7 and ABCA12 are associated with Alzheimer's disease [43]. Therefore, we can speculate that the interaction among rs1931565, rs4505578 and APOE may affect some brain functions and therefore increase the LOAD risk. Our results do not contain any of the 10 SNPs in GAB2 found in [41]. One reason is that Rieman *et al*. only explored two-locus interactions related with LOAD. In fact, the epistatic interactions are very complicated. If we restrict the number of genetic risk factors as two, we will miss some potential disease SNPs associated with complex diseases.

### Analysis of Autism Data

Autism is a common early onset neurodevelopmental disorder, which affects the brain's normal development and impairs social interaction and communication. To pinpoint the causal SNPs and genes involved in autism, a large number of genotyping data have been generated from subjects with and without autism. Some of the genotyping data have been deposited on the AGRE (Autism Genetic Resource Exchange) website http://www.agre.org for further analysis by the research community. In this paper, we analyse one of the largest genotype dataset contributed by Children's Hospital of Philadelphia (CHOP), which contains 513,312 SNPs genotyped from 1784 cases and 2441 controls [44]. To reduce the searching space and focus on more relevant SNPs, we only use SNPs in autism-related genes. We first get information of 277 autism-related genes from the autism genetic database (AGD) http://wren.bcf.ku.edu/[45]. Then we obtain a list of 205,589 SNPs in these autism-related genes from UCSC genome browser http://genome.ucsc.edu/[46]. The CHOP dataset contains 9330 of these 205,589 SNPs. Samples with missing rate > 10% and SNPs with missing rate > 10%, MAF < 0.05, and p-value of HWE < 0.001 were removed. After applying the aforementioned filtering process, our genotype dataset contains 4222 samples (1783 cases, 2439 controls) and 8198 SNPs.

Heterogeneity of phenotypic presentation in autism makes it difficult to detect epistatic interactions related with this complex disorder [47]. One proposed approach to reduce the phenotypic heterogeneity of autistic subjects is dividing them into several subgroups by clustering method on ADI-R (Autism Diagnostic Interview-Revised) data [48]. The ADI-R is a clinical diagnostic interview to assess autism in children and adults and contains 93 items about behaviours in three domains: quality of social interaction, communication and language ability, and repetitive, restricted and stereotyped interests and behaviour [49]. In this paper, we use an alternative method to reduce the phenotypic heterogeneity: biclustering [50]. Biclustering methods can identify subgroups of autism samples that show similar behaviour patterns on a specific subset of ADI-R items. By applying the biclustering method [50], we find a bicluster of constant value in 235 subjects for 8 out of 77 numerically scored ADI-R items (0 = normal; 3 = most severe). These autistic subjects have the same ADI-R score (i.e., 3 which is most severe) on the 8 ADI-R items which are: CCONVER, CINAPPQ, CPRON, CNEOID, CVERRIT, CINR, CSPEECH, and CFRIEND. Most of these 8 items are about verbal and nonverbal communication. Therefore these 235 autism samples may represent a subset with the most severe communication problems.

To explore the genetic basis in the identified more homogeneous subset, we use the SNP data for these 235 autistic subjects (cases) and 2439 controls in CHOP dataset. The MCMC method first selects 111 candidate SNPs. Then our EpiBN detects an epistatic interaction of three SNPs: rs706363, rs7780487, and rs12536378. The first SNP, rs706363, is on the autism candidate gene DAB1 on chromosome 1. Both rs7780487 and rs12536378 are on the autism candidate gene DPP6 on chromosome 7. If we search HPRD (Human Protein-protein Interaction Database), we can find a pathway from DAB1 to DPP6: DAB1--APLP2--PRNP--DPP6 [51]. This suggests a potential interaction between the detected SNPs using our EpiBN, which warrants further investigations to assess this in silico prediction by molecular functional experiments.

## Discussion

Jiang *et al*. also tried to use score-based Bayesian network structure learning methods to detect epistatic interactions [52]. They evaluated the performance of 22 BN scoring criteria by scoring all 1-SNP, 2-SNP, 3-SNP, and 4-SNP Bayesian networks on simulation datasets and showed that the BDeu score with large values of hyperparameters *α *achieved the best performance. Since the prior knowledge on the optimal *α *and the true number of disease SNPs is unknown, their methods can hardly address the two critical challenges (small sample sizes and high dimensionality) in epistatic interaction detection very well.

## Conclusions

To address the two critical challenges (small sample sizes and high dimensionality) in epistatic interaction detection from GWAS data, several machine learning or statistical methods have been proposed during the past decade. However, these proposed machine learning or statistical methods still encounter some problems: scalability to real genome-wide dataset, tending to introduce false positives, sample-efficiency, and poor performance when detecting epistatic interactions with weak or no marginal effects.

In this paper, we propose a Bayesian network-based method, EpiBN, to detect epistatic interactions. We develop a new scoring function, which can reflect higher-order epistatic interactions by estimating the model complexity from data, and apply a fast B&B algorithm to learn the structure of a two-layer Bayesian network containing only one target node. To make our method scalable to GWAS data, we propose the use of a MCMC method to perform the screening process.

We apply the proposed method to both simulated datasets based on four disease models and three real datasets. Our experimental results demonstrate that our method outperforms some other commonly-used methods and is scalable to GWAS data.

## Competing interests

The authors declare that they have no competing interests.

## Authors' contributions

BH designed and implemented the EpiBN method, tested the existing methods and analyzed experimental results. XWC conceived the study, designed the experiments and analyzed the results. ZT contributed in autism data analysis and assisted with analyzing experimental results. HX discussed the methods and analyzed some of the results. All authors helped in drafting the manuscript and approved the final manuscript.
